# Supplementary material for: Identification of photoperiod-induced specific miRNAs in the adrenal glands of Sunite sheep (Ovis aries)
Source: Front Vet Sci. 2022 Jul 22;9:888207. doi: 10.3389/fvets.2022.888207 (PMC9354845; doi:10.3389/fvets.2022.888207)
Supplement: Supplementary file 1 [file Data_Sheet_1.ZIP › Supplementary file/miRDeep2 file.zip!/miRDeep2 file/Know_Structure/SPLP42A3/oar-mir-200a.pdf]

miRBase precursor : oar-mir-200a
Total read count : 112
oar-miR-200a read count : 105
remaining reads : 7

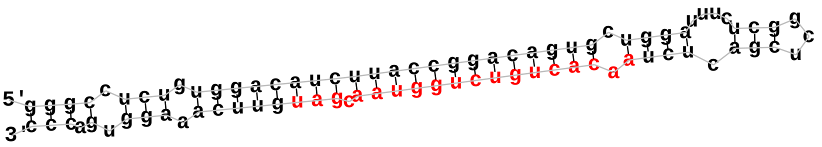

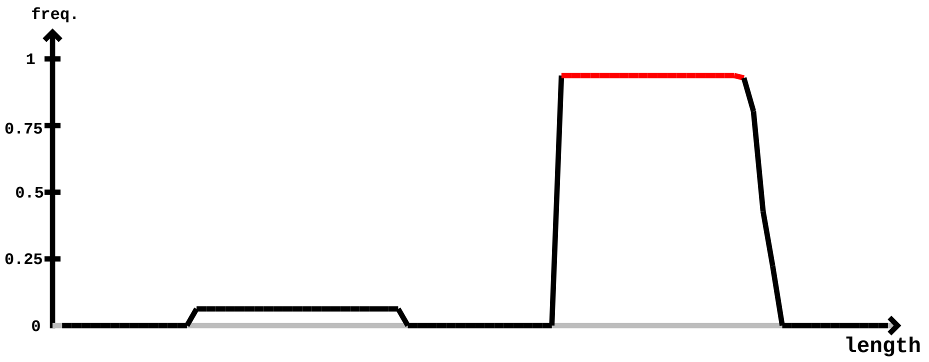

oar-miR-200a

5'-	gggccucuguggacaucuuaccggacagugcuggauuucucggcucgacucuaacacugucugguaacgauguucaaaggugaccc	-3'	exp	
	((((.(((.(((((((((((((((((((((.((((....(((...))).)))).))))))))))))).)))))))).))).).)))	reads	mm	sample
	.............caucuuaccggacagugcugga...	7	0	seq
	....uaacacugucugguaaAga................	1	1	seq
	....uaacacugucugguaacgau...............	2	0	seq
	....uaacacugucugguaaAgau...............	12	1	seq
	....uaacacugucugguaaAgaug..............	13	1	seq
	....uaacacugucugguaacgaug..............	29	0	seq
	....uaacacugucugguaacgaugu.............	23	0	seq
	....uaacacugucugguaacgauguu............	25	0	seq
